# Optimizing irrigation scheduling for winter wheat using the AquaCrop model in Xinjiang, China

**DOI:** 10.3389/fpls.2025.1680820

**Published:** 2025-10-10

**Authors:** Qiuping Fu, Xueru Liu, Ming Hong, Pengrui Ai, Liang Ma, Jinghua Zhao, Quanjiu Wang

**Affiliations:** ^1^ College of Water Conservancy and Civil Engineering, Xinjiang Agricultural University, Urumqi, China; ^2^ Xinjiang Future Irrigation District Engineering Technology Research Center, Wensu, China; ^3^ National Key Laboratory of Water Engineering Ecological Environment in Arid Areas, Xi’an University of Technology, Xi’an, China

**Keywords:** irrigation schedule, aquacrop model, winter wheat, entropy weight method, irrigation decision

## Abstract

**Introduction:**

Establishing an appropriate irrigation schedule is fundamental for the sustainable management of agricultural water resources, effectively alleviating water scarcity and ensuring regional food security.

**Methods:**

In this study, the AquaCrop model was calibrated and validated using field experimental data of winter wheat collected from 2022 to 2024. Irrigation schedules for three typical rainfall years—wet, normal, and dry—were determined, and a multi-objective optimization approach was proposed by coupling the AquaCrop model with the entropy weight method.

**Results:**

The results showed that the AquaCrop model accurately simulated canopy cover, aboveground biomass, soil water storage, and yield of winter wheat. To achieve the maximum yield, 15, 16, and 18 irrigation events were required in wet, normal, and dry years, respectively, with an irrigation quota of 30 mm per event and a lower soil water content threshold maintained at 50% of readily available water (RAW). In contrast, when the objective shifts from maximizing yield to maximizing water use efficiency (WUE), the highest WUE was achieved with 3, 4, and 5 irrigations in wet, normal, and dry years, respectively, with RAW thresholds of 90%, 90%, and 80%, and an irrigation quota of 80 mm. When considering multi-objective optimization to minimize irrigation water while maximizing yield and WUE, the recommended irrigation schedules were 3 irrigations for wet years and 4 irrigations for both normal and dry years, with RAW thresholds of 90%, 90%, and 110%, respectively, and an irrigation quota of 80 mm.

**Discussion:**

The findings provide a theoretical basis and technical support for developing optimized irrigation schedules and making informed irrigation decisions for winter wheat in arid regions.

## 1 Introduction

Wheat is the second most widely produced cereal crop globally, and winter wheat accounts for approximately 75% of the total wheat cultivation area, exerting a significant influence on global food security (Wang and Zhang, 2020). Xinjiang, located in an arid and semi-arid region, is one of the most important agricultural production areas in China (Wang et al., 2012). In 2024, the winter wheat planting area in Xinjiang reached 883,000 hectares, with a total output of 7.03 million tons (Ma et al., 2023). However, the region receives an average annual precipitation of only 270 mm, while the annual surface evaporation is about 1,000 mm. Agriculture accounts for 93.2% of total water consumption in Xinjiang, with agricultural water use reaching 51.37 billion m^3^ in 2017, and the proportion in southern Xinjiang exceeding 96% (Wang et al., 2021). Persistent drought and severe water scarcity have become critical constraints on the sustainable development of agriculture in this region (Li et al., 2020). Therefore, developing appropriate irrigation schedules and improving water use efficiency (WUE) are essential to promoting sustainable agricultural development in Xinjiang.

Traditionally, irrigation schedules have been determined through field experiments or theoretical calculation methods. However, field experiments are time-consuming and labor-intensive, and their results often have limited applicability for large-scale promotion (Shen et al., 2020). Theoretical approaches are usually based on the water balance principle, in which reference evapotranspiration is commonly estimated using the Penman–Monteith equation and then multiplied by a crop coefficient (K_c_) to determine actual crop evapotranspiration. However, accurately determining K_c_ is challenging (Nie et al., 2024), and when water stress occurs during crop growth, evapotranspiration calculated using the Penman–Monteith equation often exceeds the actual values (Fernández, 2023). In recent years, with the continuous development of crop growth models, they have been widely applied for irrigation scheduling. Representative models include WOFOST (de Wit et al., 2019, 2012), DSSAT (Dettori et al., 2017; Porter et al., 2010), APSIM (Chimonyo et al., 2016; Li et al., 2009), and AquaCrop (Foster et al., 2017), each differing in modeling principles and focus, while AquaCrop focuses on soil-water-driven crop growth and yield formation, requiring fewer input parameters and offering a user-friendly interface, making it particularly suitable for analyzing the relationship between soil moisture and yield and for water management applications (Yang et al., 2022; Guo et al., 2018; Ran et al., 2017).

The AquaCrop model simulates crop yield through canopy cover and harvest index under different management practices and irrigation scheduling, evaluating the yield response to water availability via WUE (Zhang et al., 2017; Wang et al., 2015). AquaCrop has been applied to various crops such as rice, wheat, and maize to develop efficient irrigation strategies that maximize water productivity while maintaining high yields (Sharafkhane et al., 2024). For instance, Gadédjisso-Tossou et al. (2020) simulated maize growth under full and deficit irrigation in the semi-arid regions of West Africa, while Boulange et al. (2025) applied AquaCrop to simulate cotton growth in the semi-arid climate of Uzbekistan. Amiri et al. (2014) compared CERES-Rice, AquaCrop, and ORYZA2000 models for rice under different irrigation timings and nitrogen levels and found that AquaCrop provided more accurate yield estimates. Singh et al. (2013) simulated wheat growth under flood irrigation in West Bengal, India, with good agreement between simulated and observed yields. In northern China, Du et al. (2011) used AquaCrop to simulate winter wheat biomass and yield under drip, sprinkler, and flood irrigation, achieving model efficiency indices above 0.95, with drip irrigation showing the highest simulation accuracy. Unlike the light-driven DSSAT model or the CO_2_-driven WOFOST model, AquaCrop is a water productivity-driven model developed by FAO that calculates daily soil-water balance, making it more suitable for assessing the impacts of water availability on crop yield (Raes et al., 2009a; Steduto et al., 2009). Abedinpour (2021) simulated wheat growth under varying water and nitrogen levels using DSSAT-CERES and AquaCrop and recommended AquaCrop due to its lower data requirements and reliable yield estimation. Although AquaCrop has been widely used to optimize irrigation scheduling for winter wheat, most studies have focused on single objectives such as minimizing irrigation water, maximizing yield, or maximizing WUE. Few studies have comprehensively considered multi-objective optimization that simultaneously balances irrigation water use, yield, and WUE, which is critical for improving irrigation decision-making in water-scarce regions.

Therefore, the objectives of this study were to: (i) calibrate and validate the AquaCrop model parameters using winter wheat field experiments conducted from 2022 to 2024; (ii) evaluate the impacts of different irrigation scenarios on winter wheat yield and WUE using the AquaCrop model; (iii) couple the AquaCrop model with the entropy weight method to comprehensively assess irrigation scenarios based on irrigation water use, yield, WUE, and to identify the optimal irrigation schedule for winter wheat in the study area.

## 2 Materials and methods

### 2.1 Study area description

**Figure 1:**
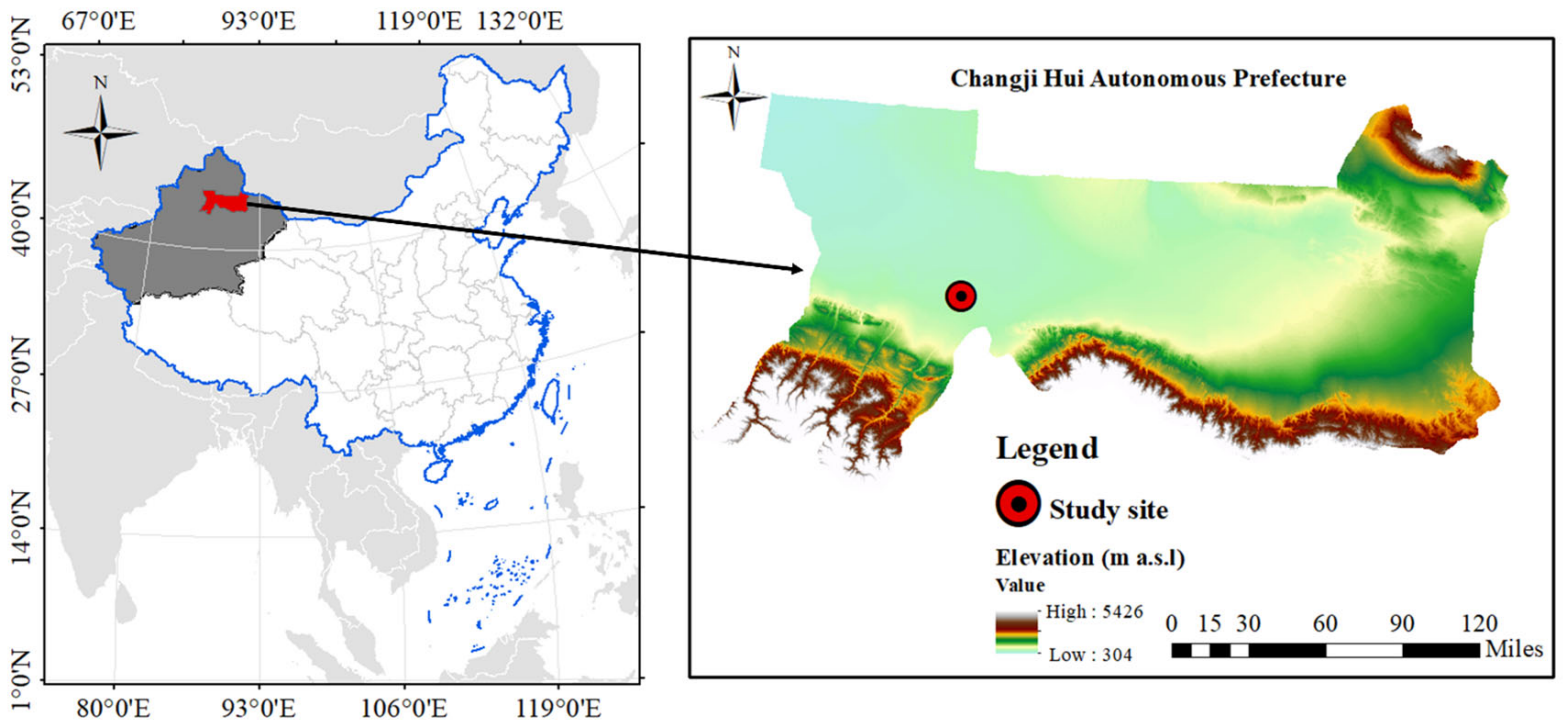
The location of experimental site.

Field experiments on winter wheat were conducted over two growing seasons from September 2022 to June 2024 at Huaxing Farm, Changji City, Changji Hui Autonomous Prefecture, Xinjiang, China (44°15′22″N, 87°15′34″E) (**Figure 1**). The study area is characterized by a typical inland desert climate, with a mean annual temperature of 6.6 °C, an average annual precipitation of 280 mm, a mean annual evaporation of 1,787 mm, and a total annual sunshine duration of 2,833 h.

### 2.2 Data collection

#### 2.2.1 Meteorological data

**Figure 2:**
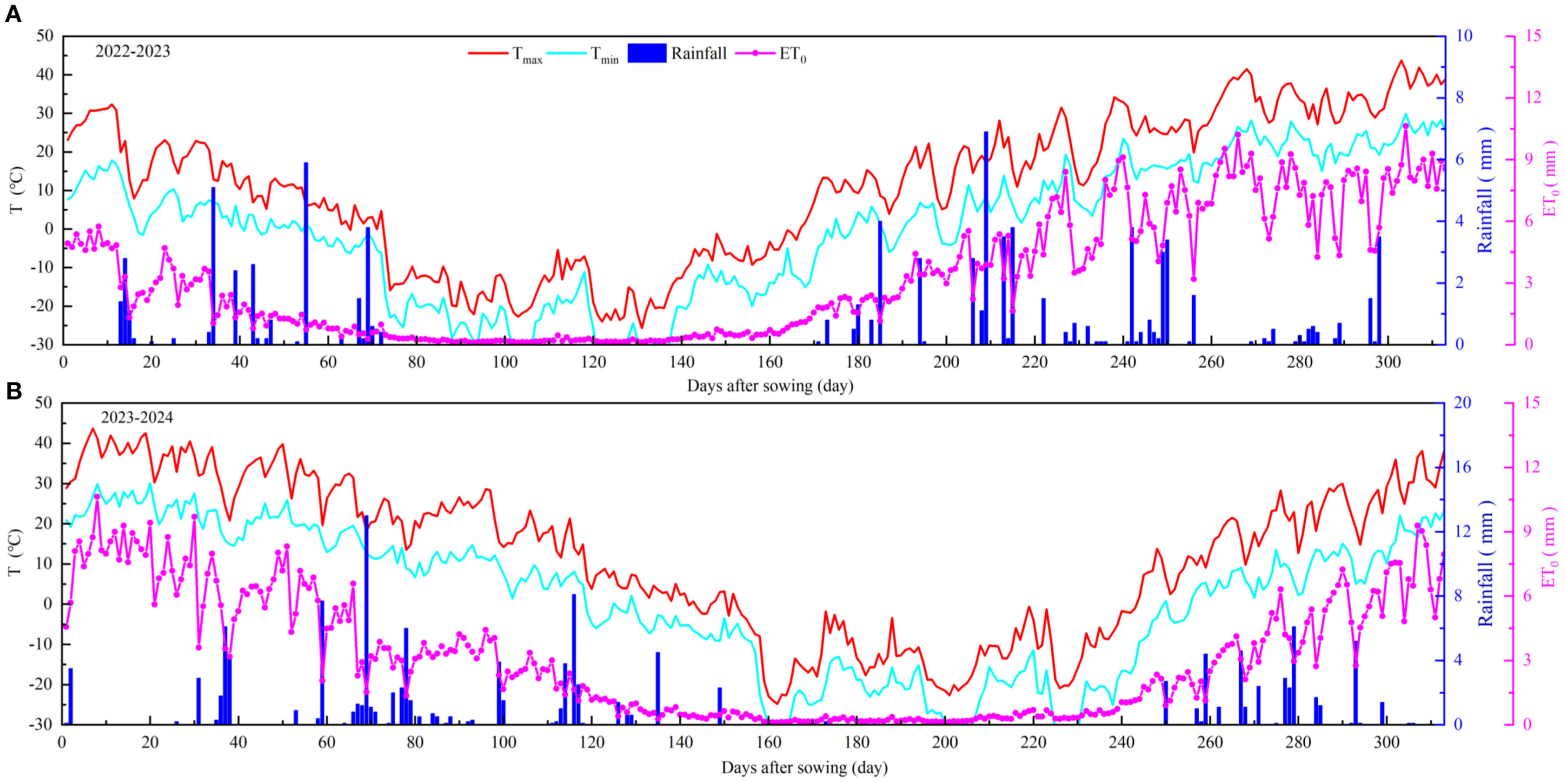
Daily maximum and minimum temperature (T_max_ and T_min_), rainfall, and reference evapotranspiration (ET_o_) in winter wheat growing seasons of 2022-2024.

Meteorological data were collected from an on-site automatic weather station (HOBO U30, USA) installed at the experimental site, which continuously monitored air temperature, relative humidity, solar radiation, atmospheric pressure, wind speed, wind direction, and precipitation. During the experimental period (September 2022 to July 2024), daily precipitation (Rain), maximum and minimum air temperatures (T_max_ and T_min_), and reference evapotranspiration (ET_0_) are shown in **Figure 2**. The ET_0_ during the winter wheat growing season was calculated using the FAO-56 Penman–Monteith equation, as modified by Allan et al. (1998) and Raes (2009b), with the aid of the FAO ET_0_ Calculator. The evapotranspiration file (.ET0), rainfall file (.PLU), temperature file (.TMP), and the default atmospheric CO_2_ concentration file (GlobalAverage.CO_2_) (Keeling and Whorf, 2005) were combined to generate the climate input file (.CLI) required by the AquaCrop model.

#### 2.2.2 Crop data

The winter wheat cultivar used in this study was “XinDong 22”, with a planting density of 450,000 plants ha^−1^. Sowing dates were 16 September 2022 and 12 September 2023, respectively. Wheat was sown in rows with an equal row spacing of 15 cm and a seeding depth of 3 cm. Harvesting was carried out on 9 July 2023 and 25 June 2024. For the 2022–2023 growing season, seedling emergence occurred 10 days after sowing (DAS 10). The leaf area index (*LAI*) increased steadily until the jointing stage (DAS 226), reached maximum canopy cover at the heading stage (DAS 248), and then gradually declined during flowering (DAS 259), followed by senescence in the late grain-filling stage (DAS 272). Physiological maturity occurred 300 days after sowing. For the 2023–2024 growing season, emergence, maximum canopy development, onset of senescence, and maturity were observed at DAS 8, DAS 230, DAS 246, DAS 256, DAS 270, and DAS 288, respectively.

#### 2.2.3 Management data

From September 2022 to July 2023 and from September 2023 to June 2024, the experiments were arranged in a completely randomized design, with each plot covering an area of 120 m^2^. Based on local farmers’ production practices, a drip irrigation system was adopted, and the irrigation schedules are presented in **Table 1**. Irrigation during the winter wheat growing seasons started on 11 April 2023 and 22 April 2024, respectively. Fertilization rates were consistent across both seasons: 227 kg ha^−1^ of urea (N ≥ 46%), 195 kg ha^−1^ of monoammonium phosphate (P_2_O_5_ + N ≥ 55%), and 150 kg ha^−1^ of potassium chloride (K_2_O ≥ 60%). Monoammonium phosphate and potassium chloride were applied as a basal fertilizer before sowing, while urea was top-dressed in three split applications. Each treatment was replicated three times. Three rows of buffer plants were established around the experimental field to minimize border effects. Other pest, disease, and weed management practices followed local high-yield field management standards.

**Table 1:** The irrigation schedule in the 2022–2024 entire wheat growing season, respectively.

Treatment	Irrigation period (day)	Irrigation frequency	Irrigating amount (mm)
T1	5	11	270
T2			330
T3			390
T4			450
T5	7	8	270
T6			330
T7			390
T8			450
T9	9	6	270
T10			330
T11			390
T12			450

#### 2.2.4 Soil data

Before sowing winter wheat, ten sampling points were randomly selected within the experimental field. Soil samples were collected using a soil auger at seven depth intervals: 0–10, 10–20, 20–30, 30–40, 40–60, 60–80, and 80–100 cm. The samples were sealed in polyethylene bags, oven-dried, and analyzed to determine soil bulk density, field capacity, saturated water content, and initial soil water content (**Table 2**). Soil bulk density and initial water content were measured by cutting ring method and drying method, respectively. The cutting ring that contained the soil sample was placed in a container of pure water for 24 h to slowly absorb water and saturate it, before being removed to determine the soil saturated water content. Subsequently, the saturated cores were placed on dry soil to allow gravitational water to drain, and the soil field capacity was then measured. According to the USDA Soil Taxonomy, the soil texture of the experimental site was classified as silty loam. The two experimental seasons were conducted on the same plots, and the stratified mean soil parameters for each plot are listed in **Table 2**. The chemical properties of the topsoil (0–20 cm) were as follows: soil organic matter 11.3 g kg^−1^, available nitrogen 58.4 mg kg^−1^, available phosphorus 24.4 mg kg^−1^, and available potassium 229 mg kg^−1^. The measured soil physical and chemical properties were incorporated into the AquaCrop model to generate the soil input file (*.SOL).

**Table 2:** The soil hydraulic parameters in the experimental field.

Soil texture	Depth (cm)	Bulk density (g m^−3^)	*θ_s_* (cm^3^ cm^−3^)	*θ_fc_* (cm^3^ cm^−3^)	*θ_pwp_* (cm^3^ cm^−3^)	*θ*_0_ (cm^3^ cm^−3^)
Silty loam	0-10	1.42	0.44	0.36	0.08	0.24
	10-20	1.41	0.45	0.37	0.08	0.26
	20-30	1.45	0.45	0.36	0.08	0.28
	30-40	1.41	0.45	0.37	0.10	0.26
	40-60	1.39	0.49	0.40	0.10	0.27
	60-80	1.33	0.49	0.40	0.12	0.24
	80-100	1.41	0.49	0.41	0.12	0.26

*θ_s_* represents saturated soil water content; *θ_fc_* represents field capacity; *θ_pwp_* represents permanent wilting point; *θ*_0_ represents initial soil water content.

#### 2.2.5 Canopy cover, aboveground biomass, soil water storage and yield

During the jointing, heading, flowering, grain-filling, and maturity stages of winter wheat, soil samples were collected from seven depth intervals: 0–10 cm, 10–20 cm, 20–30 cm, 30–40 cm, 40–60 cm, 60–80 cm, and 80–100 cm. The gravimetric soil water content of each layer was determined using the oven-drying method. Based on the gravimetric water content, bulk density, and soil layer thickness, the soil water storage (*SWS*, mm) in the 0–100 cm profile was calculated using the following Equation (1):


(1) $$SWS=10\times \theta_{m}\times BD\times H$$

where *θ_m_* is the gravimetric soil water content (cm^3^ cm^−3^), *BD* is the soil bulk density (g cm^−3^), and *H* is the thickness of the soil layer (cm).

During the jointing, heading, flowering, grain-filling, and maturity stages of winter wheat, ten representative plants were selected from each plot. For each plant, the maximum length and width of all green leaves were measured, and the *LAI* was calculated according to Equation (2) Heng et al. (2018):


(2) $$LAI=0.75\times \rho \times \frac{\sum_{j=1}^{m}\left(L_{nj}\times W_{nj}\right)}{m}$$

where *ρ* is the planting density (plants m^−2^), *m* is the number of sampled plants, *L_nj_* is the maximum length of the j-th leaf (cm), *W_nj_* is the maximum width of the j-th leaf (cm), 0.75 is the leaf shape coefficient.

Canopy cover (*CC*) represents the degree of crop canopy development, expressed as the proportion of green leaf area covering the plot surface. It was derived from the *LAI* and calculated according to Equation (3) (Katerji et al., 2013):


(3) $$CC=1.005\times {\left(1-{\text{e}}^{-0.6LAI}\right)}^{1.2}$$

During the jointing, heading, flowering, grain-filling, and maturity stages of winter wheat, ten representative plants were selected from each plot. The aboveground plant samples were first oven-dried at 105 °C for 30 min to deactivate enzymes and then dried at 80 °C to a constant weight to determine the aboveground biomass. At maturity, uniform areas within each plot were selected to measure yield components, including the number of spikes per meter in two adjacent rows, the number of grains per spike, and the thousand-grain weight. For yield determination, a 3 m^2^ area was harvested from each plot, threshed, and weighed to record the yield.

#### 2.2.6 Water use efficiency

WUE was defined as the yield obtained per unit of water consumed and was calculated according to Equation (4) (Tan et al., 2018):


(4) $$WUE=\frac{Y}{10\times ET}\times 1000$$

where *Y* is the yield (t ha^−1^), and *ET* is simulated evapotranspiration during the entire growing season (mm). The *ET* was estimated using the soil water balance method for winter wheat according to Equation (5) (Fernández et al., 2020):


(5) $$ET=\Delta W+I+P+S_{g}-D-R$$

where *ΔW* is the change in soil water storage between sowing and harvest (mm), *I* is the irrigation amount (mm), *P* is the precipitation (mm), *S_g_* is the groundwater recharge (mm), *D* is the deep percolation (mm), and *R* is the surface runoff (mm).

### 2.3 Model evaluation

The AquaCrop model was calibrated by data obtained in the first growing season (2022–2023) and validated using data from the second growing season (2023–2024). Evaluation of the model performance was based on the model’s accuracy in simulating canopy cover, aboveground biomass, soil water storage, and yield. Several statistical indicators were used to compare model performance based on each calibration scenario. Root-mean square error (*RMSE*), Willmott’s index of agreement (*d*), and coefficient of determination (*R* ^2^) were obtained according to Equations (6–8).


(6) $$RMSE=\sqrt{\frac{\sum_{i=1}^{n}{\left(S_{i}-M_{i}\right)}^{2}}{n}}$$

(7) $$d=1-\frac{\sum_{i=1}^{n}{\left(S_{i}-M_{i}\right)}^{2}}{\sum_{i=1}^{n}{\left(|S_{i}-\bar{S}|+|M_{i}-\bar{M}|\right)}^{2}}$$

(8) $$R^{2}=\frac{{\left[\sum_{i=1}^{n}{\left(M_{i}-\bar{M}\right)}^{2}(S_{i}-\bar{S})\right]}^{2}}{\sum_{i=1}^{n}{\left(M_{i}-\bar{M}\right)}^{2}\sum_{i=1}^{n}{\left(S_{i}-\bar{S}\right)}^{2}}$$

where is *M_i_*, *S_i_*, and $\bar{M}$ are the measured value, simulated value, and average value of measurements. Values of *R* ^2^ and *d* close to 1 indicate the model’s good performance. *RMSE* values close to zero indicate a good matching between simulated values and observations.

### 2.4 Irrigation scheduling scenarios

To investigate the effects of irrigation schedules under typical rainfall years on winter wheat yield and WUE in Xinjiang, and to optimize irrigation management for high winter wheat productivity, multiple irrigation scenarios were designed. The scenario design followed the framework of Shao et al. (2018), primarily considering two factors: soil water content at the time of irrigation and irrigation quota. The soil water content scenarios were set to cover the full range of water stress levels that winter wheat might experience during its growing season in the study region, from the lowest to the highest water availability. The irrigation quotas encompassed the minimum and maximum feasible field application amounts. In the model, soil water content was expressed as a percentage of readily available water (RAW), where RAW is defined as half the difference between field capacity and the wilting point. Volumetric soil moisture levels corresponding to 50%, 60%, 70%, 80%, 90%, 100%, 110%, and 120% of RAW were simulated. Drip irrigation was employed with irrigation quotas set at 30, 40, 50, 60, 70, and 80 mm, resulting in a total of 48 combined irrigation scenarios.

Due to the arid climate and low precipitation in the study area, insufficient soil moisture at sowing leads to water deficit, which is a critical factor limiting seedling emergence. Therefore, a pre-sowing irrigation event was applied to ensure uniform emergence across all scenarios. The water demand from sowing to mid-April was met by soil water stored from non-seasonal rainfall and pre-sowing irrigation event (Moghbel et al., 2024). For rational irrigation scheduling, irrigation events were assumed to start 223 days after sowing. Based on local planting and harvesting dates, the sowing date was set to 10 September and the harvest date to 1 July.

This study employed the Pearson-III distribution to analyze rainfall data during the winter wheat growing seasons from 1990 to 2020 in the study area. Based on this analysis, typical hydrological years were selected according to their exceedance probabilities: wet years (25% exceedance probability), normal years (50%), and dry years (75%) (Guo et al., 2021). Three representative years were chosen for simulation to assess the irrigation-induced yield potential of winter wheat: 1997–1998 (177 mm) as a wet year, 1999–2000 (134.8 mm) as a normal year, and 2005–2006 (98.5 mm) as a dry year.

### 2.5 Entropy weight method

The entropy weight method is a multi-criteria decision-making approach that determines indicator weights based on the degree of variation in the information contained within each indicator. It uses information entropy to describe the relative rate of change in sample data: the closer the coefficient is to 1, the nearer it is to the optimal target, whereas a coefficient closer to 0 indicates a greater deviation from the target (Elena Arce et al., 2015). In the function construction, each irrigation scenario is denoted as the *k*-th scenario, while the irrigation amount, yield, and WUE under different irrigation scenarios are represented as the *j*-th indicator of the *k*-th scenario. Among these indicators, higher yield and WUE are preferable, whereas lower irrigation amounts are considered better. The function can thus be formulated as follows:

(1) Processing of original indicator data:

Positive indicators were obtained according to Equation (9):


(9) $$x_{kj}^{'}=x\frac{x_{kj}-x_{\min}}{x_{\max}-x_{\min}}$$

Negative indicators were obtained according to Equation (10):


(10) $$x_{kj}^{'}=\frac{x_{\max}-x_{j}}{x_{\max}-x_{\min}}$$

(2) Standardization eliminates the dimensional differences of indicators were obtained according to Equation (11):


(11) $$x_{kj}^{"}=\frac{x_{kj}^{'}-\mu_{j}}{\sigma_{j}}\;(1\leq k\leq m,\;1\leq j\leq n)$$

where *μ_j_* is the mean value of indicator *j*, *σ_j_* is the standard deviation of indicator *j*, x″ is the standardized value of the indicator, *m* is the total number of irrigation scenarios (48 in this study), and *n* is the number of evaluation indicators, namely irrigation amount, yield, and WUE. Since the standardized values may include negative numbers, a translation (shift) is applied to ensure that all standardized values are positive before subsequent entropy calculations according to Equation (12):


(12) $$Z_{kj}=x_{kj}^{"}-\min(x_{j}^{"})+\epsilon$$

where *ϵ* is a very small constant (10^−6^) added to avoid zero values in the entropy calculation.

(3) The proportion of the *k*-th irrigation scenario under the *j*-th indicator, denoted as *P_kj_*, was calculated according to Equation (13):


(13) $$P_{kj}=\frac{Z_{kj}}{\sum_{k=1}^{m}Z_{kj}}$$

(4) Calculation of the entropy value *e_j_* for indicator *j* was carried out according to Equation (14):


(14) $$e_{j}=-\frac{1}{m}\sum_{j=1}^{m}p_{kj}\text{ In}p_{kj}$$

(5) Calculation of the weight *ω_j_* for indicator *j* was carried out according to Equation (15):


(15) $$\omega_{j}=\frac{1+\frac{1}{m}\sum_{k=1}^{m}p_{kj}\text{In}p_{kj}}{\sum_{j=1}^{n}(1+\frac{1}{m}\sum_{k=1}^{m}p_{kj}\text{In}p_{kj})}$$

(6) Calculation of the comprehensive evaluation score *F_k_* for the *k*-th irrigation scenario was carried out according to Equation (16):


(16) $$F_{k}=\sum_{j=1}^{n}\omega_{j}p_{kj}$$

where *ω_j_* is the weight of the *j*-th indicator, *p_kj_* is the normalized value of the *j*-th indicator under the *k*-th irrigation scenario, and *n* is the total number of indicators (irrigation amount, yield, and WUE in this study). A higher *F_k_* indicates a more favorable irrigation scenario when considering multiple objectives simultaneously.

## 3 Results

### 3.1 Model parameter calibration

Determining crop parameters—including canopy development, aboveground biomass accumulation, yield formation parameters, and responses to water, temperature, and salinity stress—is essential for calibrating the AquaCrop model. Following the winter wheat parameters recommended in the AquaCrop model manual (Raes et al., 2009a), the model was calibrated using the observed data collected during the 2022–2023 winter wheat growing season. The main crop parameters used in the model are listed in **Table 3**. The canopy growth coefficient (*CGC*), canopy decline coefficient (*CDC*), normalized biomass water productivity (WP*), and reference harvest index (*HI*_0_) are key parameters controlling canopy dynamics, aboveground biomass accumulation, and yield formation in AquaCrop, and are generally considered constant for a given crop cultivar (Steduto et al., 2009). However, due to differences in cultivar characteristics and local climatic conditions, adjustments within the recommended parameter ranges are acceptable (Hsiao et al., 2009).

**Table 3:** Main crop parameters in AquaCrop for winter wheat under drip irrigation.

Symbol	Description	Value	Unit	Remarks
Parameters of canopy development and production				
*CC*_0_	Initial canopy cover	4.5	%	Measured
*CC_x_*	Maximum canopy cover	96	%	Measured
*CGC*	Canopy-growth coefficient	3.2	% d^−1^	Calibrated
*CDC*	Canopy-decline coefficient	10.2	% d^−1^	Calibrated
*Zr_min_*	Minimum effective rooting depth	0.1	m	Measured
*Zr_max_*	Maximum effective rooting depth	1.2	m	Measured
*f_shape,z_*	Shape factor for root-zone expansion	1.5	–	Recommended
*Kc_Tr_*	Crop coefficient at *CC* = 100% prior to senescence	1.1	–	Recommended
*f_yield_*	Water productivity normalized for ET_o_ and CO_2_ during yield formation	72	%	Calibrated
*f_CO_*_2_	Crop performance under elevated atmospheric CO_2_ concentration	60	%	Calibrated
*S_m_*	Maximum root-water extraction over effective root zone	44	mm day^−1^	Measured
WP^*^	Water productivity normalized for ET_o_ and CO_2_	17	g m^−2^	Calibrated
*HI_o_*	Reference harvest index	42	%	Calibrated
Parameters of water-stress response				
*P_exp,upper_*	Fraction of TAW at which canopy expansion is limited	0.1	–	Calibrated
*P_exp,lower_*	Fraction of TAW at which canopy expansion stops	0.45	–	Calibrated
*P_exp,shp_*	Shape factor for water-stress coefficient of canopy expansion	3.5	–	Recommended
*P_sto,upper_*	Fraction of TAW at the beginning of stomatal closure	0.45	–	Calibrated
*f_shape,sto_*	Shape factor for water-stress coefficient of stomatal closure	2.0	–	Recommended
*P_sen,upper_*	Fraction of TAW at the beginning of early canopy senescence	0.6	–	Calibrated
*f_shape,sen_*	Shape factor for water-stress coefficient of canopy senescence	2.0	–	Recommended
*P_pol_*	Fraction of TAW at the beginning of pollination failure	0.85	–	Recommended
Parameters of air temperature stress response				
*T_base_*	Base temperature	0	°C	Calibrated
*T_upper_*	Upper temperature	40	°C	Calibrated
*GD_upper_*	The upper threshold at which crop transpiration will be limited by air temperature	5	°C day	Calibrated
Parameters of salinity-stress response				
*ECe_lower_*	*EC_e_* at which crop starts to be affected	3	dS m^−1^	Calibrated
*Ece_upper_*	*EC_e_* at which crop can no longer grow	14	dS m^−1^	Calibrated
*CCD*	Canopy deformation degree	25	%	Recommended
*f_ks,sto,salt_*	Shape factor for the response of stomatal closure	100	%	Recommended

In this study, the calibrated *CGC* and *CDC* values were 3.2% d^−1^ and 10.2% d^−1^, respectively. Compared to the recommended values for winter wheat (4.9% d^−1^ for *CGC* and 7.2% d^−1^ for *CDC*), *CGC* was lower while *CDC* was higher. The calibrated WP* and *HI*_0_ were 17 g m^−2^ and 42%, respectively. WP* was slightly higher than the recommended value of 15 g m^−2^, while *HI*_0_ was lower than the recommended value of 48%, but both remained within the suggested ranges of 15–20 g m^−2^ and 25–50%. In addition, the calibrated yield formation adjustment factor (*f*_yield_) was 72%, lower than the default value of 100%. The sink strength coefficient under elevated CO_2_ concentrations (*f*_CO2_) was calibrated to 60%, which is higher than the recommended value of 50% for wheat but still within the reasonable range of 40–60%.

For the water, temperature, and salinity stress parameters, several key thresholds were adjusted within the recommended ranges of the AquaCrop model manual to better represent the local field conditions. For water stress, the soil water depletion thresholds were set to 0.10–0.45 for canopy expansion, 0.45 for stomatal conductance, and 0.60 for canopy senescence. For temperature stress, the base temperature, upper temperature limit, and upper growing-degree threshold (*GD*_upper_) were adjusted to 0 °C, 40 °C, and 5 °C day, respectively. For salinity stress, the salinity threshold affecting crop growth was set to 3–14 dS m^−1^. All these parameters were tuned within the recommended ranges using a “method of trial and error” to ensure that the simulated results were consistent with the observed field conditions.

**Figure 3:**
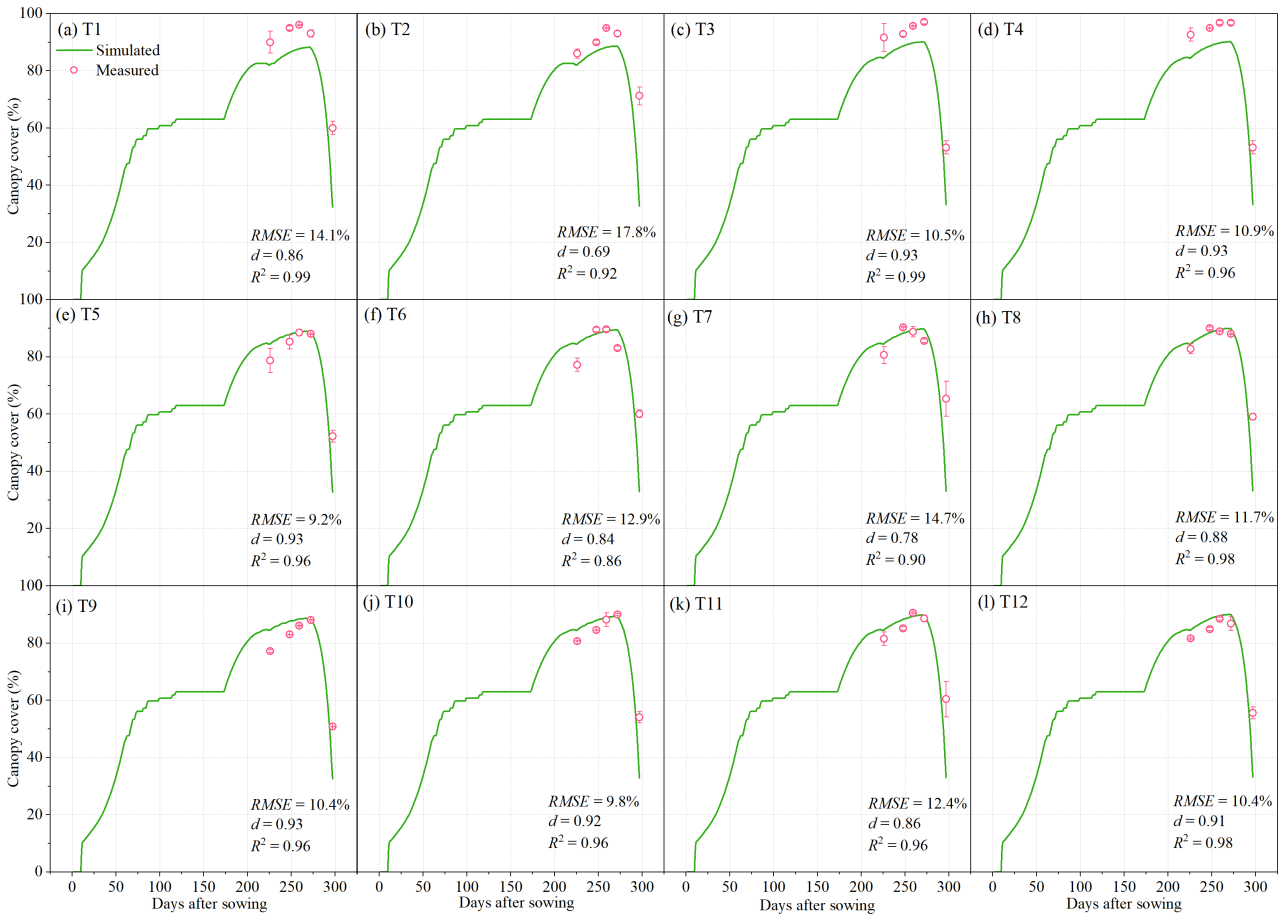
Measured and simulated canopy cover for the irrigation treatments of T1-T12. The error bars represent standard deviations.

**Figure 4:**
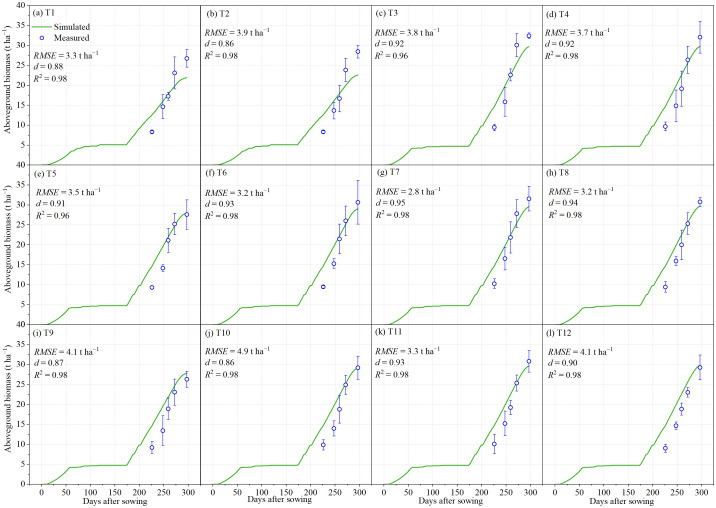
Measured and simulated aboveground biomass for the irrigation treatments of T1-T12. The error bars represent standard deviations.

**Figure 5:**
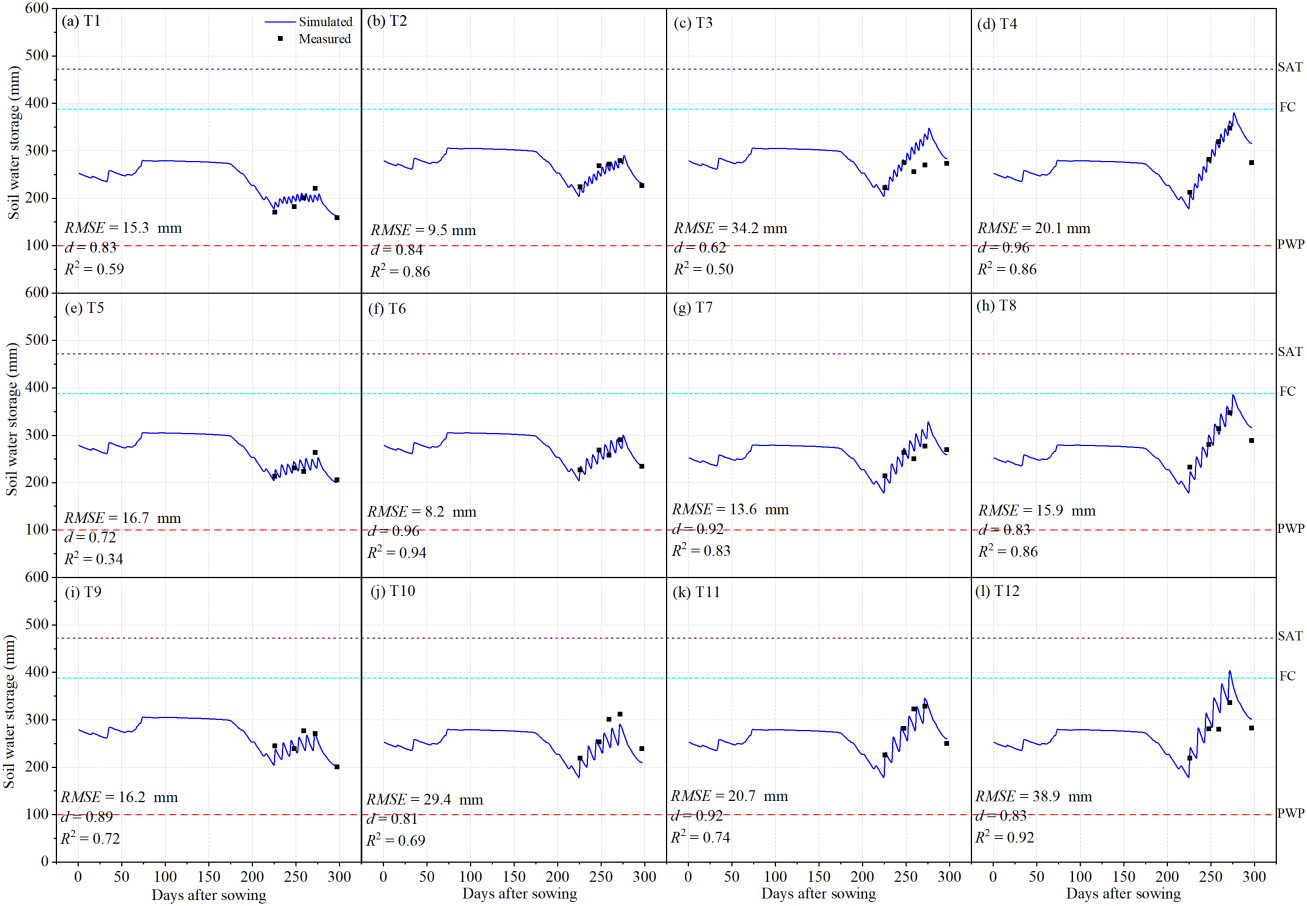
Measured and simulated soil water storage (0–100 cm) for the irrigation treatments of T1-T12. .

**Figure 6:**
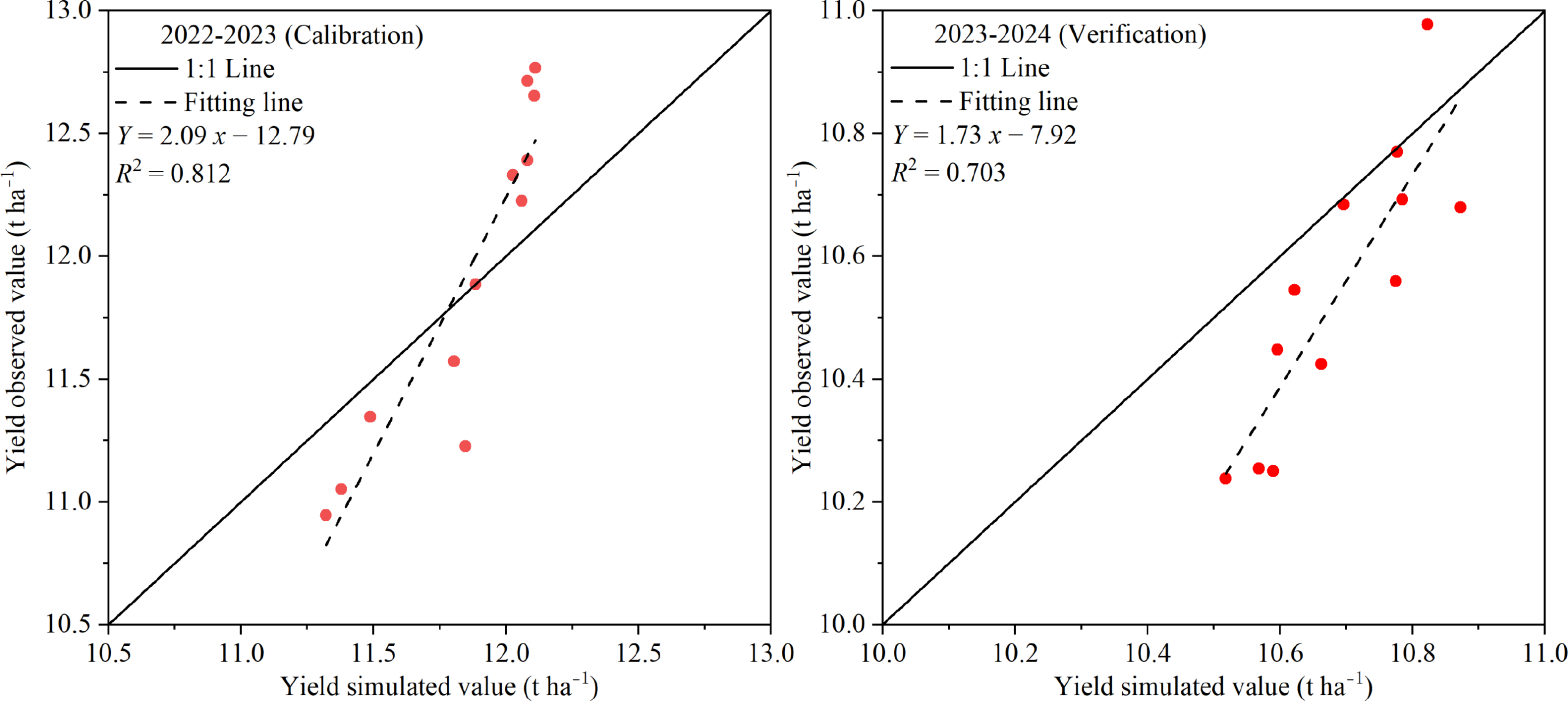
Observed and simulated yield for the irrigation treatments of T1-T12.

The AquaCrop model was calibrated using canopy cover, aboveground biomass, 0–100 cm soil water storage, and yield data obtained from all treatments during the 2022–2023 field experiment. For treatments T1–T12, the simulated canopy cover showed good agreement with field observations, with *RMSE* values ranging from 9.2% to 17.8%, *d* ranging from 0.69 to 0.93, and *R* ^2^ between 0.86 and 0.99 (**Figure 3**). Similarly, the simulated aboveground biomass closely matched the measured data, with *RMSE* values of 2.8–4.9 t ha^−1^, *d* values of 0.86–0.95, and *R* ^2^ values of 0.96–0.98 (**Figure 4**). These results indicate that the calibrated model parameters provided a reliable simulation of canopy development and biomass accumulation in winter wheat. For 0–100 cm soil water storage, the model performance was slightly lower, with *RMSE* values ranging from 8.2 mm to 38.9 mm, *d* values of 0.62–0.96, and *R* ^2^ values of 0.34–0.94 (**Figure 5**). The relatively lower accuracy for soil water storage was mainly attributed to spatial heterogeneity in soil water content within the field, which affects simulation precision compared with canopy cover and biomass. For yield, the simulated values showed a strong correlation with field measurements, with an *R* ^2^ of 0.812 (**Figure 6A**). Overall, the results demonstrate that the calibrated AquaCrop model can reasonably simulate the growth dynamics of winter wheat and the temporal variation of soil water storage in the study region. However, further validation is needed to confirm its broader applicability for winter wheat under the climatic and soil conditions of Xinjiang.

### 3.2 Model validation

After parameter calibration, the AquaCrop model was validated using field observations from the T1–T12 irrigation treatments during the 2023–2024 winter wheat growing season. As shown in **Table 4**, the simulated canopy cover closely matched the measured values, with *RMSE* values ranging from 10.3% to 14.4%, *d* ranging from 0.61 to 0.71, and *R* ^2^ between 0.94 and 0.98. The aboveground biomass of winter wheat exhibited a typical three-phase trend over time, characterized by an “increase–stagnation–increase” pattern. The simulated biomass agreed well with the measured data, with *RMSE* values of 4.7–8.5 t ha^−1^, *d* values of 0.76–0.87, and *R* ^2^ values of 0.83–0.98. The underlying mechanism is as follows: After emergence and before overwintering, rapid leaf growth led to an expansion of canopy cover, resulting in high photosynthetic assimilation and a steady increase in aboveground biomass. During the overwintering stage, when daily mean temperatures dropped below 0 °C, wheat entered a dormant or semi-dormant state. Cell division and elongation nearly ceased, and the activity of photosynthetic enzymes was inhibited, causing biomass accumulation to stagnate. Following the jointing stage, when the daily mean temperature exceeded 5 °C, wheat resumed active growth. Photosynthetic enzyme activity was restored, leaf photosynthetic rates increased rapidly, and assimilates were redistributed from leaves to stems, sheaths, and spikes, leading to accelerated biomass accumulation.

**Table 4:** AquaCrop model verification results.

Treatment	Canopy cover (%)			Aboveground biomass (t ha^−1^)			Soil water storage (mm)		
	*RMSE*	*d*	*R* ^2^	*RMSE*	*d*	*R* ^2^	*RMSE*	*d*	*R* ^2^
T1	14.1	0.62	0.96	6.7	0.80	0.96	18.7	0.88	0.72
T2	10.3	0.71	0.98	6.8	0.76	0.95	12.6	0.96	0.86
T3	11.6	0.67	0.96	5.2	0.87	0.98	13.7	0.96	0.86
T4	11.7	0.67	0.96	6.1	0.80	0.90	14.6	0.95	0.88
T5	13.6	0.63	0.98	8.5	0.76	0.96	30.2	0.75	0.85
T6	11.7	0.67	0.96	6.6	0.80	0.98	30.9	0.81	0.67
T7	11.5	0.68	0.96	4.7	0.81	0.83	30.2	0.73	0.55
T8	11.1	0.68	0.96	7.6	0.79	0.96	20.2	0.91	0.71
T9	14.0	0.61	0.94	7.2	0.78	0.98	19.6	0.92	0.76
T10	13.1	0.64	0.96	5.3	0.86	0.92	27.0	0.81	0.50
T11	13.7	0.63	0.96	5.6	0.83	0.96	22.4	0.85	0.79
T12	10.8	0.69	0.96	5.4	0.82	0.96	28.6	0.78	0.49

For 0–100 cm soil water storage, the model showed reasonable performance, with *RMSE* values of 12.6–30.9 mm, *d* values of 0.73–0.96, and *R* ^2^ values of 0.49–0.88. For yield, the simulated values exhibited a moderate correlation with field measurements, with an *R* ^2^ of 0.703 (**Figure 6B**). It may be attributed to the spatial heterogeneity of soil properties, differences in field management practices, and some unconsidered environmental stress factors (such as pests and diseases, lack of nutrition) that affect actual yield. Overall, these results indicate that, based on canopy cover, aboveground biomass, soil water storage, and yield observations from the 2023–2024 experimental data, the validated AquaCrop model demonstrated satisfactory accuracy, which is acceptable for practical irrigation management applications. Thus, the calibrated and validated AquaCrop model can reliably simulate winter wheat growth characteristics and soil water dynamics under the climatic and soil conditions of the study region.

### 3.3 Irrigation schedule optimization

**Figure 7:**
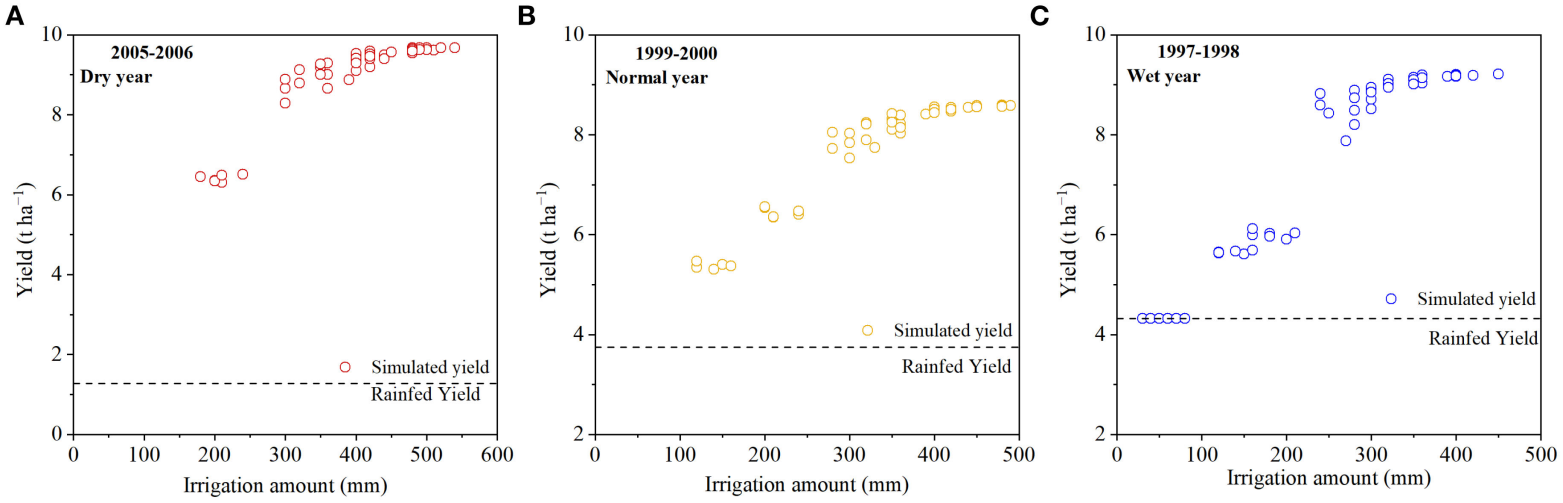
Winter wheat yield under different irrigation schedules in dry year **(A)**, normal year **(B)**, and wet year **(C)**.

Optimizing winter wheat irrigation scheduling under different rainfall scenarios requires an understanding of how yield responds to irrigation amount. **Figure 7** illustrates the simulated relationships between irrigation depth and winter wheat yield under 48 irrigation scenarios for three typical rainfall years. As shown in **Figure 7**, the response of winter wheat yield to irrigation followed two distinct phases: Rapid increase phase–At lower irrigation levels, yield increased sharply with additional irrigation, indicating that water availability was the primary limiting factor for crop growth. Yield stabilization phase–Once irrigation reached a certain threshold, further increases in irrigation had only marginal effects, with yields stabilizing at a high level or showing only slight additional gains. In dry years, limited in-season rainfall resulted in severe water deficits. Under rainfed conditions, the yield was only 1.275 t ha^−1^, but supplemental irrigation of 540 mm increased the yield to 9.677 t ha^−1^ (**Figure 7A**). This corresponds to an average yield gain of 1.556 t ha^−1^ for every additional 100 mm of irrigation. In normal years, the rainfed yield was 3.746 t ha^−1^, while supplemental irrigation of 480 mm increased the yield to 8.589 t ha^−1^ (**Figure 7B**), equivalent to 1.009 t ha^−1^ yield gain per 100 mm of irrigation. In wet years, the rainfed yield was relatively higher (4.324 t ha^−1^). With 450 mm of supplemental irrigation, yield reached 9.214 t ha^−1^ (**Figure 7C**), resulting in 1.087 t ha^−1^ yield gain per 100 mm of irrigation. These results clearly indicate that the potential yield response to irrigation is highest in drought years, followed by wet years, and lowest in normal years. This pattern arises because, in drought years, water is the primary limiting factor, so supplemental irrigation produces the largest yield gains. In wet years, rainfall partially mitigates water stress, leading to a moderate yield response. In normal years, rainfall generally meets crop water requirements, so additional irrigation contributes only minimally to yield increases.

**Tables 5**, **6**, **7** present the simulated yield and WUE of winter wheat under 48 irrigation scenarios for wet, normal, and dry years. The results show that the highest-yielding irrigation scheduling required 15, 16, and 18 irrigation events in wet, normal, and dry years, respectively, with an irrigation depth of 30 mm per event and a readily available water (RAW) of 50%, representing the lower control limit of RAW. In contrast, the highest WUE was achieved with only 3, 4, and 5 irrigation events in wet, normal, and dry years, respectively, with higher soil water depletion thresholds (RAW of 90%, 90%, and 80%) and a larger irrigation depth of 80 mm per event.

**Table 5:** Optimized irrigation schedules in wet year.

Scenario	*F*	Rank	*RAW*	Irrigating quota (mm)	Irrigating times	Irrigation amount (mm)	Yield (t ha^−1^)	WUE (kg m^−3^)
W1	0.0193	36	50%	30	15	450	9.214	1.67
W2	0.0206	29	60%		13	390	9.162	1.75
W3	0.0211	25	70%		12	360	9.037	1.78
W4	0.0213	23	80%		10	300	8.512	1.77
W5	0.0207	27	90%		9	270	7.879	1.7
W6	0.0189	42	100%		6	180	6.022	1.5
W7	0.0195	32	110%		4	120	5.646	1.52
W8	0.0190	40	120%		1	30	4.324	1.44
W9	0.0207	26	50%	40	10	400	9.209	1.78
W10	0.0207	28	60%		10	400	9.162	1.78
W11	0.0220	16	70%		8	320	8.972	1.85
W12	0.0218	18	80%		7	280	8.490	1.81
W13	0.0215	21	90%		7	280	8.198	1.79
W14	0.0194	33	100%		4	160	5.997	1.54
W15	0.0195	31	110%		3	120	5.629	1.53
W16	0.0188	43	120%		1	40	4.324	1.44
W17	0.0211	24	50%	50	8	400	9.193	1.83
W18	0.0219	17	60%		7	350	9.149	1.87
W19	0.0225	8	70%		6	300	8.877	1.89
W20	0.0221	14	80%		6	300	8.708	1.86
W21	0.0224	11	90%		5	250	8.427	1.84
W22	0.0184	47	100%		4	200	5.908	1.48
W23	0.0189	41	110%		3	150	5.612	1.5
W24	0.0187	44	120%		1	50	4.324	1.44
W25	0.0221	15	50%	60	6	360	9.190	1.9
W26	0.0217	19	60%		6	360	9.136	1.86
W27	0.0228	6	70%		5	300	8.948	1.92
W28	0.0225	9	80%		5	300	8.847	1.89
W29	0.0231	4	90%		4	240	8.592	1.9
W30	0.0190	38	100%		3	180	5.966	1.52
W31	0.0194	34	110%		2	120	5.641	1.51
W32	0.0186	45	120%		1	60	4.324	1.44
W33	0.0214	22	50%	70	6	420	9.187	1.9
W34	0.0222	13	60%		5	350	9.099	1.91
W35	0.0222	12	70%		5	350	9.010	1.92
W36	0.0231	2	80%		4	280	8.892	1.94
W37	0.0226	7	90%		4	280	8.737	1.89
W38	0.0190	39	100%		3	210	6.029	1.56
W39	0.0193	35	110%		2	140	5.664	1.53
W40	0.0185	46	120%		1	70	4.324	1.44
W41	0.0215	20	50%	**80**	5	400	9.178	1.89
W42	0.0231	3	60%		4	320	9.113	1.98
W43	0.0229	5	70%		4	320	9.025	1.96
W44	0.0225	10	80%		4	320	8.945	1.91
W45	**0.0239**	1	**90%**		**3**	**240**	**8.819**	**1.99**
W46	0.0202	30	100%		2	160	6.121	1.64
W47	0.0192	37	110%		2	160	5.687	1.55
W48	0.0184	48	120%		1	80	4.324	1.44

*F* represents the assessment value, *RAW* represents readily available soil water, and *WUE* represents water use efficiency. Bold values indicate the top-ranked irrigation scenario.

**Table 6:** Optimized irrigation schedules in normal year.

Scenario	*F*	Rank	*RAW*	Irrigating quota (mm)	Irrigating times	Irrigation amount (mm)	Yield (t ha^−1^)	WUE (kg m^−3^)
N1	0.0182	46	50%	30	480	16	8.589	1.52
N2	0.0188	41	60%		450	15	8.55	1.53
N3	0.0208	27	70%		390	13	8.412	1.6
N4	0.0203	31	80%		360	12	8.032	1.58
N5	0.0197	35	90%		330	11	7.742	1.55
N6	0.0198	34	100%		300	10	7.534	1.55
N7	0.0182	45	110%		210	7	6.343	1.49
N8	0.0179	47	120%		120	4	5.348	1.48
N9	0.0191	39	50%	40	480	12	8.579	1.56
N10	0.0202	32	60%		440	11	8.543	1.59
N11	0.0210	23	70%		400	10	8.45	1.61
N12	0.0214	20	80%		360	9	8.217	1.62
N13	0.0209	26	90%		320	8	7.895	1.59
N14	0.0216	19	100%		280	7	7.719	1.61
N15	0.0199	33	110%		200	5	6.535	1.55
N16	0.0184	43	120%		120	3	5.339	1.5
N17	0.0209	25	50%	50	450	9	8.581	1.63
N18	0.0207	29	60%		450	9	8.559	1.62
N19	0.0214	21	70%		400	8	8.436	1.63
N20	0.0225	10	80%		350	7	8.315	1.66
N21	0.0216	18	90%		350	7	8.108	1.63
N22	0.0219	13	100%		300	6	7.833	1.63
N23	0.0203	30	110%		200	4	6.558	1.57
N24	0.0178	48	120%		150	3	5.398	1.49
N25	0.0207	28	50%	60	480	8	8.577	1.64
N26	0.0217	17	60%		420	7	8.548	1.65
N27	0.0218	15	70%		420	7	8.472	1.66
N28	0.0227	7	80%		360	6	8.388	1.67
N29	0.0217	16	90%		360	6	8.147	1.64
N30	0.0226	9	100%		300	5	8.028	1.65
N31	0.0188	40	110%		240	4	6.401	1.53
N32	0.0196	36	120%		120	2	5.47	1.55
N33	0.0212	22	50%	70	490	7	8.581	1.67
N34	0.0219	14	60%		420	6	8.549	1.66
N35	0.0225	11	70%		420	6	8.504	1.69
N36	0.0233	4	80%		350	5	8.422	1.69
N37	0.0224	12	90%		350	5	8.247	1.66
N38	0.0237	2	100%		280	4	8.045	1.69
N39	0.0193	38	110%		210	3	6.354	1.54
N40	0.0185	42	120%		140	2	5.307	1.52
N41	0.0209	24	50%	**80**	480	6	8.563	1.65
N42	0.0228	6	60%		400	5	8.55	1.69
N43	0.0229	5	70%		400	5	8.511	1.7
N44	0.0226	8	80%		400	5	8.437	1.69
N45	**0.0238**	**1**	**90%**		**320**	**4**	**8.236**	**1.71**
N46	0.0234	3	100%		320	4	8.209	1.69
N47	0.0195	37	110%		240	3	6.466	1.56
N48	0.0183	44	120%		160	2	5.371	1.52

*F* represents the assessment value, *RAW* represents readily available soil water, and *WUE* represents water use efficiency. Bold values indicate the top-ranked irrigation scenario.

**Table 7:** Optimized irrigation schedules in dry year.

Scenario	*F*	Rank	*RAW*	Irrigating quota (mm)	Irrigating times	Irrigation amount (mm)	Yield (t ha^−1^)	WUE (kg m^−3^)
D1	0.0174	45	50%	30	540	18	9.677	1.64
D2	0.0187	42	60%		510	17	9.619	1.70
D3	0.0193	38	70%		480	16	9.539	1.71
D4	0.0197	37	80%		420	14	9.194	1.71
D5	0.0192	40	90%		390	13	8.868	1.68
D6	0.0199	36	100%		360	12	8.662	1.71
D7	0.0201	35	110%		300	10	8.288	1.70
D8	0.0170	46	120%		210	7	6.31	1.6
D9	0.0192	39	50%	40	520	13	9.674	1.73
D10	0.0201	34	60%		480	12	9.640	1.75
D11	0.0208	28	70%		440	11	9.490	1.77
D12	0.0210	26	80%		440	11	9.394	1.79
D13	0.0207	32	90%		400	10	9.096	1.76
D14	0.0210	27	100%		360	9	9.009	1.75
D15	0.0216	18	110%		320	8	8.795	1.77
D16	0.0167	48	120%		200	5	6.353	1.57
D17	0.0205	33	50%	50	500	10	9.678	1.79
D18	0.0208	29	60%		500	10	9.632	1.81
D19	0.0211	25	70%		450	9	9.562	1.79
D20	0.0221	15	80%		400	8	9.486	1.81
D21	0.0214	21	90%		400	8	9.338	1.78
D22	0.0222	11	100%		350	7	9.181	1.80
D23	0.0223	10	110%		300	6	8.654	1.80
D24	0.0169	47	120%		200	4	6.337	1.58
D25	0.0215	20	50%	60	480	8	9.678	1.83
D26	0.0207	31	60%		480	8	9.637	1.79
D27	0.0228	6	70%		420	7	9.590	1.86
D28	0.0220	17	80%		420	7	9.496	1.82
D29	0.0213	23	90%		420	7	9.401	1.79
D30	0.0226	7	100%		360	6	9.297	1.82
D31	0.0232	3	110%		300	5	8.883	1.84
D32	0.0189	41	120%		180	3	6.444	1.67
D33	0.0213	22	50%	70	490	7	9.674	1.83
D34	0.0208	30	60%		490	7	9.631	1.80
D35	0.0231	5	70%		420	6	9.593	1.88
D36	0.0221	12	80%		420	6	9.515	1.83
D37	0.0221	14	90%		420	6	9.456	1.83
D38	0.0232	4	100%		350	5	9.269	1.85
D39	0.0223	9	110%		350	5	9.007	1.82
D40	0.0181	43	120%		210	3	6.485	1.65
D41	0.0221	13	50%	**80**	480	6	9.662	1.87
D42	0.0216	19	60%		480	6	9.630	1.84
D43	0.0212	24	70%		480	6	9.590	1.82
D44	0.0237	2	80%		400	5	9.537	1.90
D45	0.0225	8	90%		400	5	9.409	1.84
D46	0.0220	16	100%		400	5	9.298	1.82
D47	**0.0241**	**1**	**110%**		**320**	**4**	**9.125**	**1.89**
D48	0.0174	44	120%		240	3	6.509	1.63

*F* represents the assessment value, *RAW* represents readily available soil water, and *WUE* represents water use efficiency. Bold values indicate the top-ranked irrigation scenario.

### 3.4 Irrigation schedule decision

By constructing a multi-objective optimization function that simultaneously minimizes irrigation volume while maximizing grain yield and water use efficiency, we calculated the comprehensive evaluation index (*F_k_*) for all 48 irrigation scenarios under wet, normal, and dry precipitation years (**Tables 5**, **6**, **7**). The results showed that the optimal irrigation schedule in wet year consisted of a soil water depletion threshold of 90% RAW, with an irrigation quota of 80 mm applied 3 times, resulting in a total irrigation volume of 240 mm, a yield of 8.819 t ha^−1^, and a WUE of 1.99 kg m^−3^. In normal year, the optimal scheduling was 90% RAW, 80 mm per application, with 3 irrigations totaling 320 mm, achieving a yield of 8.236 t ha^−1^ and a WUE of 1.71 kg m^−3^. In dry year, the optimal scheduling was 110% RAW, with 80 mm per application, applied 4 times, yielding 9.125 t ha^−1^ with a WUE of 1.89 kg m^−3^.

These results demonstrate that multi-objective optimization can effectively balance water savings and productivity, producing near-maximum yields with substantially reduced irrigation inputs compared to the yield-maximizing irrigation schedule. Notably, the optimized scheduling required only 3–4 irrigations with larger individual irrigation depths, maintaining sufficient soil moisture to avoid irreversible water stress during critical growth stages. From a practical standpoint, this approach enables flexible irrigation scheduling according to annual precipitation conditions, offering a sustainable compromise between maximizing yield, improving water productivity, and minimizing water use in water-scarce regions like Xinjiang.

## 4 Discussion

In recent years, numerous studies have demonstrated that the AquaCrop model exhibits sufficient accuracy in simulating the dynamics of canopy cover, aboveground biomass, yield, and soil water storage in winter wheat, indicating that it is a reliable and valuable tool for investigating yield and WUE responses. For example, Han et al. (2019) evaluated the applicability of AquaCrop for a winter wheat–summer maize rotation system in the North China Plain and reported normalized root mean square errors (*NRMSE*) of 15.9% for canopy cover and 4.23% for yield simulations. Similarly, Iqbal et al. (2014) tested the model’s ability to simulate winter wheat yield, biomass, actual evapotranspiration, and soil water storage (0–120 cm) in the same region, reporting *RMSE* values of 0.58 t ha^−1^ for yield, 0.87 t ha^−1^ for biomass, 33.2 mm for actual evapotranspiration, and 24.5–37.6 mm for soil water storage, with corresponding *d* values of 0.92, 0.95, 0.93, and 0.85–0.90, respectively. Furthermore, Moghbel et al. (2024) employed AquaCrop to investigate the effects of irrigation intervals and supplemental irrigation strategies on winter wheat in the U.S. Midwest, achieving *RMSE*, *NRMSE*, and *d* values of 0.85 t ha^−1^, 0.06, and 0.85 for biomass, and 0.79 t ha^−1^, 0.17, and 0.68 for yield. These findings are consistent with the results of the present study, where the model achieved high simulation accuracy for canopy cover (**Figure 3**), aboveground biomass (**Figure 4**), 0–100 cm soil water storage (**Figure 5**), and yield (**Figure 6**). Such consistency confirms that AquaCrop provides reliable simulations of winter wheat growth dynamics and soil water processes under the climatic conditions of Xinjiang. Therefore, it can be considered a robust tool for irrigation scheduling and optimization, offering valuable guidance for improving water productivity and sustaining high yields in arid and semi-arid regions.

The response of winter wheat yield to irrigation quotas under different typical hydrological years revealed clear differences among rainfall regimes. Under rainfed conditions, grain yield was highest in the wet year (4.324 t ha^−1^), followed by the normal year (3.746 t ha^−1^), and lowest in the dry year (1.275 t ha^−1^) (**Figure 7**). This pattern can be attributed to the significant influence of seasonal rainfall amount and distribution on winter wheat productivity (Wang et al., 2022). Using the AquaCrop model, optimized irrigation schedules were determined for typical hydrological years in the study region. The results showed that maximum grain yield was achieved with irrigation quotas of 450 mm (15 irrigations), 480 mm (16 irrigations), and 540 mm (18 irrigations) in wet, normal, and dry years, respectively, with a fixed irrigation depth of 30 mm per event. In contrast, the highest WUE was obtained with irrigation quotas of 240 mm, 320 mm, and 400 mm in wet, normal, and dry years, respectively, by applying fewer irrigation events (3, 4, and 5 times) with a larger irrigation depth of 80 mm per event (**Tables 5**, **6**, **7**). These findings are consistent with previous results on winter wheat irrigation management in Xinjiang (Lei et al., 2017; Yang et al., 2025). It is worth noting that the determination of optimal irrigation scheduling is closely related not only to the total rainfall but also to the intra-seasonal rainfall distribution within the wheat growth stages. Even when the total seasonal rainfall is similar across years, rainfall events occurring at different phenological stages can have markedly different impacts on crop growth and yield formation (Guo et al., 2021; Moghbel et al., 2024; Shao et al., 2018; Xing et al., 2016). Therefore, the determination of winter wheat irrigation schedule is closely related to the rainfall distribution of the selected typical hydrological year. Moreover, the uncertainty of future precipitation regimes implies that irrigation schedules derived from historical typical hydrological years should be regarded as reference scenarios rather than fixed prescriptions. In practical applications, these model-based irrigation strategies need to be flexibly adjusted according to real-time meteorological conditions, water availability, and local management practices to ensure sustainable water resource utilization and stable wheat production.

Decision-making priorities differ among stakeholders depending on their production scale and objectives. In the context of crop production, stakeholders can generally be categorized into smallholder farmers and large-scale farms, with the most notable differences lying in farm size, input intensity, and management goals. Large-scale farms operate on extensive areas with substantial capital investment, thus prioritizing maximized net economic returns. In contrast, smallholder farmers emphasize stable grain yields while minimizing time and labor inputs (Jian et al., 2018). To account for these differences, this study coupled the AquaCrop model with an entropy-based multi-objective optimization method to derive optimal irrigation schedules for winter wheat under different typical hydrological years. Based on the optimized scenarios, irrigation regimes were further averaged across hydrological conditions to provide practical irrigation recommendations tailored to farmers and large-scale farms. Under different rainfall scenarios, the optimized irrigation frequency often resulted in non-integer values, which are impractical for field implementation. Therefore, for ease of on-farm application, the irrigation frequency for smallholder farmers was rounded to 3–4 irrigations per season, with an irrigation depth of 80 mm per event, prioritizing water-saving and reduced management requirements. In contrast, large-scale farms were recommended to adopt 16–17 irrigations per season, with a smaller irrigation depth of 30 mm per event, ensuring near-optimal soil moisture conditions and maximizing yield potential. Thus, the optimized irrigation strategies for winter wheat in Xinjiang can be summarized as follows: For smallholder farmers, apply 80 mm per irrigation, 3–4 times per season, focusing on higher water productivity and reduced operational complexity. For large-scale farms, apply 30 mm per irrigation, 16–17 times per season, targeting maximum yield benefits with precise soil moisture management. These differentiated irrigation recommendations provide a flexible decision-making framework that allows stakeholders to select irrigation schedules according to their operational scale, resource availability, and production objectives.

Irrigation optimization is one of the most effective approaches for designing irrigation strategies under limited water supply, with the core objective of determining how to allocate finite water resources across different growth stages to achieve either maximum yield or maximum water productivity (Jian et al., 2018). Previous studies have typically relied on scenario-based statistical analyses of AquaCrop model simulations to optimize irrigation regimes (Li et al., 2005; Zhu et al., 2024). In this study, we coupled the AquaCrop model with a multi-objective optimization framework based on the entropy weight method, providing a novel approach for winter wheat irrigation scheduling under water-limited conditions. This method enables multi-scale decision support, offering tailored irrigation strategies for different stakeholders, including irrigation district managers, smallholder farmers, and large-scale farms, thereby enhancing both water-use efficiency and economic returns. Nevertheless, several limitations remain: (i) the optimization focused solely on water availability, without accounting for nutrient deficiencies, salinity stress, or pest and disease pressures, which may affect crop growth and yield. (ii) AquaCrop parameters were primarily calibrated using a trial-and-error approach, without conducting a formal sensitivity analysis, which could have identified the most influential parameters affecting model accuracy. (iii) the proposed optimization framework has not yet been extended to a regional-scale modeling system, limiting its applicability for large-scale water resource planning and management.

## 5 Conclusion

This study proposed a multi-objective optimization method for winter wheat irrigation scheduling and identified optimal irrigation regimes for the arid region of Xinjiang, China. The main conclusions are as follows: (i) the AquaCrop model demonstrated good accuracy in simulating the dynamic processes of canopy cover, aboveground biomass, soil water storage, and grain yield of winter wheat under different irrigation treatments in Xinjiang. (ii) the simulated results revealed the yield response potential of winter wheat to supplemental irrigation under different hydrological years. In dry years, every additional 100 mm of irrigation increased grain yield by 1.556 t ha^−1^, while in normal and wet years, the yield increase was 1.009 t ha^−1^ and 1.087 t ha^−1^, respectively. (iii) by coupling AquaCrop with the entropy weight method, a multi-objective optimization framework was developed to balance irrigation water use, yield, and WUE. The recommended irrigation schedules were 3 irrigations for wet years and 4 irrigations for both normal and dry years, with RAW thresholds of 90%, 90%, and 110%, respectively, and an irrigation quota of 80 mm.

## Data Availability

The original contributions presented in the study are included in the article/supplementary material. Further inquiries can be directed to the corresponding authors.
